# Factors associated with common mental disorders among breastfeeding mothers in tertiary hospital nurseries in Nigeria

**DOI:** 10.1371/journal.pone.0281704

**Published:** 2023-03-09

**Authors:** Michael Abel Alao, Olayinka Rasheed Ibrahim, Kenechukwu Kosisochukwu Iloh, Adaeze C. Ayuk, Udochukwu Michael Diala, Datonye Christopher Briggs, Zainab Oluwatosin Imam, Sakiru Abiodun Yekini, Sikirat Adetoun Sotimehin, Aishatu Zaidu Musa, Esther Oluwatoyin Famutimi, Adedeji Abiodun Idris, Chioma Laura Odimegwu, Zainab Kikelomo Imam, Patricia F. Medupin, Ayomide Toluwanimi Adeyemi, Kenechi Ogbodo Nnamani, Olukemi Oluwatoyin Tongo

**Affiliations:** 1 Department of Pediatrics, College of Medicine University of Ibadan & University College Hospital, Ibadan, Oyo State, Nigeria; 2 Department of Paediatrics, Federal Medical Centre, Kastina, Kastina State, Nigeria; 3 Department of Pediatrics, University of Ilorin Teaching Hospital, Ilorin, Kwara State, Nigeria; 4 Department of Paediatrics, University of Nigeria &University of Nigeria Teaching Hospital, Ituku/Ozalla, Enugu, Nigeria; 5 Department of Pediatrics, College of Health Sciences, University of Jos, Jos, Plateau State, Nigeria; 6 Rivers State University, Faculty of Clinical Sciences, College of Medical Sciences / Department of Paediatrics, Rivers State University Teaching Hospital, Port Harcourt, Rivers State, Nigeria; 7 Department of Pediatrics, Lagos State University Teaching Hospital, Lagos, Nigeria; 8 Paediatrics Department, Asokoro District Hospital / Faculty of Clinical Sciences, College of Health Sciences, Nile University of Nigeria, Abuja, Federal Capital Territory, Abuja, Nigeria; 9 Department of Paediatrics Abubakar Tafewa Balewa University, Bauchi, Bauchi State, Nigeria; 10 Department of Clinical Nursing, University College Hospital, Ibadan, Oyo State, Nigeria; 11 Women’s Mental Health Division Sidra Medicine Al Gharafa, Doha, Qatar; 12 Department of Paediatrics, Federal Medical Centre, Lokoja, Nigeria; 13 Department of Paediatrics, College of Medicine/University College Hospital Ibadan Centre for African Newborn Health and Nutrition, University College Hospital, Ibadan, Oyo State, Nigeria; 14 Department of Paediatrics, Nnamdi Azikiwe University Teaching Hospital, Nnewi, Anambra State, Nigeria; University of Abuja Teaching Hospital, NIGERIA

## Abstract

**Background:**

Several studies have shown that the impact of maternal mental health disorders on newborns’ well-being in low and middle-income countries (LMIC) are underreported, multi-dimensional and varies over time and differs from what is reported in high-income countries. We present the prevalence and risk factors associated with common mental disorders (CMDs) among breastfeeding mothers whose infants were admitted to Nigerian tertiary care facilities.

**Methods:**

This was a national cross-sectional study involving mothers of hospitalised babies from eleven Nigerian tertiary hospitals. We used the WHO self-reporting Questionnaire 20 and an adapted WHO/UNICEF ten-step breastfeeding support package to assess mothers’ mental health and breastfeeding support.

**Results:**

Only 895 of the 1,120 mothers recruited from eleven tertiary healthcare nurseries in six geopolitical zones of Nigeria had complete datasets for analysis. The participants’ mean age was 29.9 ± 6.2 years. One in four had CMDs; 24.0% (95% CI: 21.235, 26.937%). The ages of mothers, parity, gestational age at delivery, and length of hospital stay were comparable between mothers with and those without CMDs. Antenatal care at primary healthcare facilities (adjusted odds ratio [aOR:13], primary education [aOR:3.255] living in the south-southern region of the country [aOR 2.207], poor breastfeeding support [aOR:1.467], polygamous family settings [aOR:2.182], and a previous history of mental health disorders [aOR:4.684] were significantly associated with CMDs. In contrast, those from the middle and lower socioeconomic classes were less likely to develop CMDs, with [aOR:0.532] and [aOR:0.493], respectively.

**Conclusion:**

In Nigeria, the prevalence of CMDs is relatively high among breastfeeding mothers with infants admitted to a tertiary care facility. Prior history of mental illness, polygamous households, mothers living in the southern region and low or no educational attainment have a greater risk of developing CMDs. This study provides evidence for assessing and tailoring interventions to CMDs among breastfeeding mothers in neonatal nurseries in LMIC.

## Introduction

The World Health Organisation defines a mental disorder as a clinically substantial impairment in an individual’s cognitive, emotional control, or behaviour [1]. Common mental disorders (CMDs) include depression, generalised anxiety disorder, panic disorder, phobias, social anxiety disorder, obsessive-compulsive disorder, and posttraumatic stress disorder. The presence of CMD is linked to discomfort or impairment in key areas of function [1–4].

Recent years have seen an increase in the importance of mental health diseases, particularly CMD [5, 6]. In the global disease burden report, mental health disorders were included as one of the top 10 major causes of morbidity and mortality [6, 7]. The COVID-19 pandemic has been observed to exacerbate trends because of its influence on rising social isolation and other socioeconomic concerns [7–9]. According to a WHO report, approximately 1 billion people suffer from mental disorders, costing the global economy $2.5 billion per year. The global distribution of mental health disorders disproportionately affects low-middle income settings, which bear a greater burden [6, 10, 11] Unfortunately, the female gender, which appears to be more vulnerable in low-resource settings, is the most affected [6, 10, 11]. The perinatal period (pregnancy and postnatal) has a greater mental disease burden, with the highest incidence occurring in the immediate postpartum period of a woman’s life [7]. This becomes more concerning if the mother is faced with the additional task of caring for her newborn in a level 2–4 facility [10, 12–14].

A mother’s poor mental health can limit her ability to care for her child [10, 13, 14]. The consequences of a mother’s mental illness will affect her ability to support the family not only in terms of food supply but also in terms of acceptable and hygienic food provision, as well as her ability to use existing resources to meet a child’s nutritional needs [10, 13, 14] Common maternal mental health problems have been connected to 21–49% of childhood malnutrition and neonatal failure to thrive [13, 14]. Malnutrition results from poor mental health because it affects the child’s diet [14]. In addition, a mother needs to be mentally fit to produce adequate amounts of breastmilk, achieve appropriate positioning and attachment and establish effective bonding [15, 16]. Therefore, maternal mental illness often results in short- and long-term effects on growth and cognitive development [11].

It is worth noting that the relationship between a mother’s mental health and breastfeeding practice appears to be bidirectional [15, 16]. Breastfeeding has been documented as a stressor for some mothers who struggle with establishing adequate lactation or are asked to exclusively breastfeed their babies [17]. In a retrospective study, some mothers in remission from mental disorders reported a relapse as a result of the demand for exclusive breastfeeding [18]. In contrast, many prospective studies and pooled summary effects from meta-analyses show that adequate and well-supported breastfeeding is protective against mental illnesses with fewer relapses of mental breakdown in individuals with preexisting mental disorders [18].

Given the higher prevalence of mental disorders, it is critical to identify risk factors for CMD in nursing mothers [8, 9]. Identifying CMD risk factors in the context of a specific setting will allow for targeted evidence-based mental health promotion, especially in a country such as Nigeria, with a paucity of data on CMD among nursing mothers. Thus, we aimed to determine the prevalence of CMD and associated factors in mothers whose infants were admitted to Nigerian tertiary health nurseries.

## Methods

### Study design and settings

**Fig 1:**
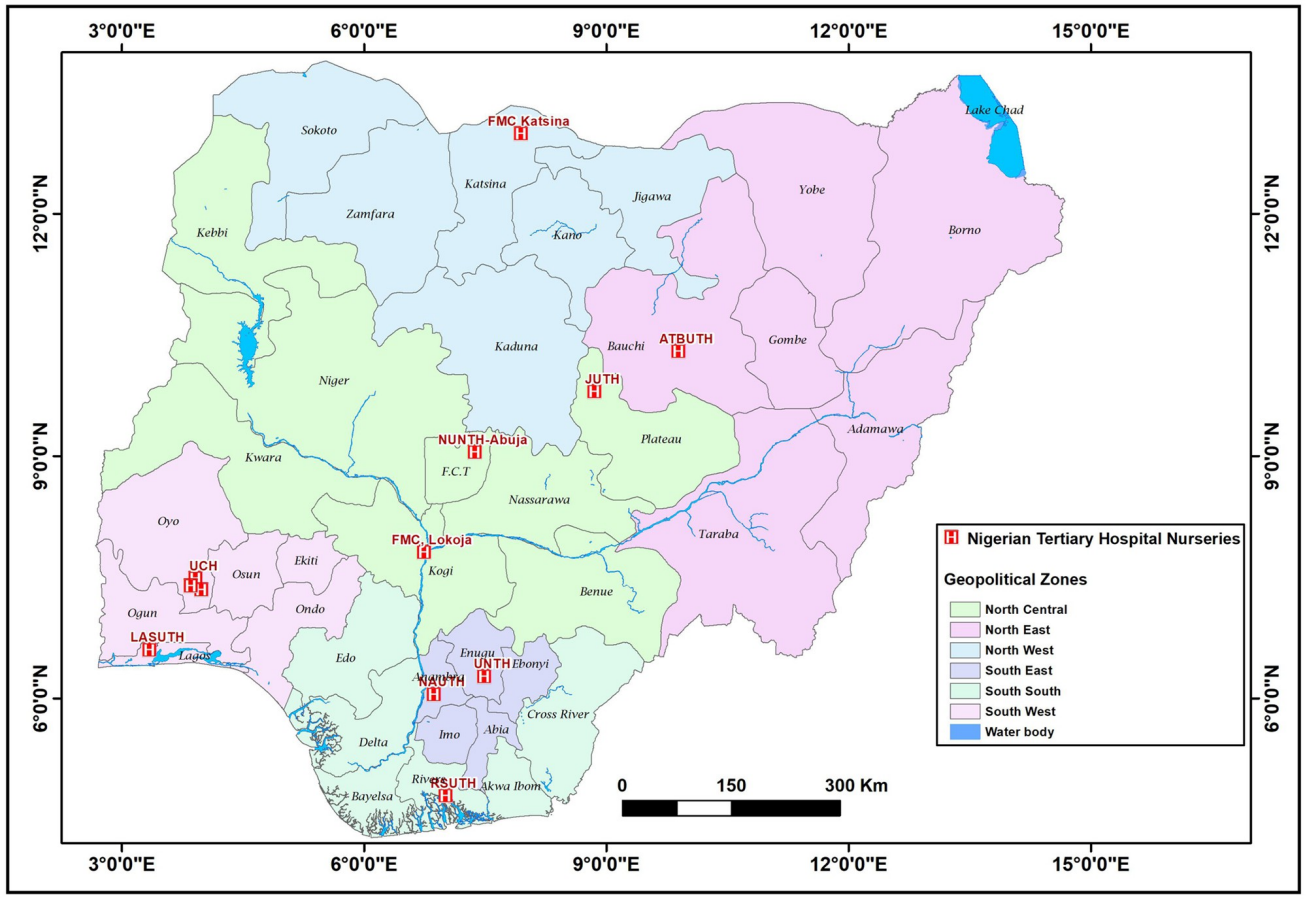
Map of Nigeria showing selected Nigerian tertiary hospital nurseries in the six geopolitical zones [UCH-University College Hospital, UITH- FMC-Katsina: Federal Medical Centre, Kastina, JUTH- Jos University Teaching Hospital, RSUTH-Rivers State University Teaching Hospital, ATBUTH-Abubakar Tafawa Balewa Teaching Hospital, Bauchi, UNTH-University of Nigeria Teaching Hospital, FMC-Lokoja; Federal Medical Centre, Lokoja, NUTH-Nile University Teaching Hospital Abuja; (formerly Asokoro District Hospital), LASUTH-, Lagos State University Teaching Hospital, NAUTH-Nnamdi Azikiwe University Teaching Hospital].

This study is part of a larger prospective, cross-sectional study conducted between May and August 2022 in twelve tertiary hospitals across Nigeria’s six geopolitical zones, including at least one hospital from each zone (Fig 1). In Nigeria, the nursery units of public tertiary hospitals have the highest bed capacity and staff strength, and they often account for the highest number of newborn hospitalizations in each state.

### Study participants

This study included suitable mother-baby dyads whose infants were hospitalised in selected Nigerian tertiary hospitals. We excluded mothers who did not provide informed consent, mothers whose babies were only fed infant formula for medical reasons, mothers who were critically ill and needed hospitalisation, mothers whose babies were admitted for less than 24 hours, and babies with multiple congenital abnormalities, surgical abdomen, or any other contraindication for oral feeds.

### Sample size determination

The minimum sample size required for this study was calculated using the ‘Raosoft software’ (http://www.raosoft.com/samplesize.html). Using a previously determined estimated 24.3% prevalence of common mental disorder among breastfeeding mothers at 95% confidence level, 3% (0.03) tolerable margin of error, and a 10% non-response rate, a sample size of 832 was obtained [19]. Due to the multisite nature of this study, the total sample size was divided by six to give a target of 80–140 mothers-dyad per geopolitical zone to the proportion of neonatal admissions.

### Subjects’ enrolment and conduct of the research

**Fig 2:**
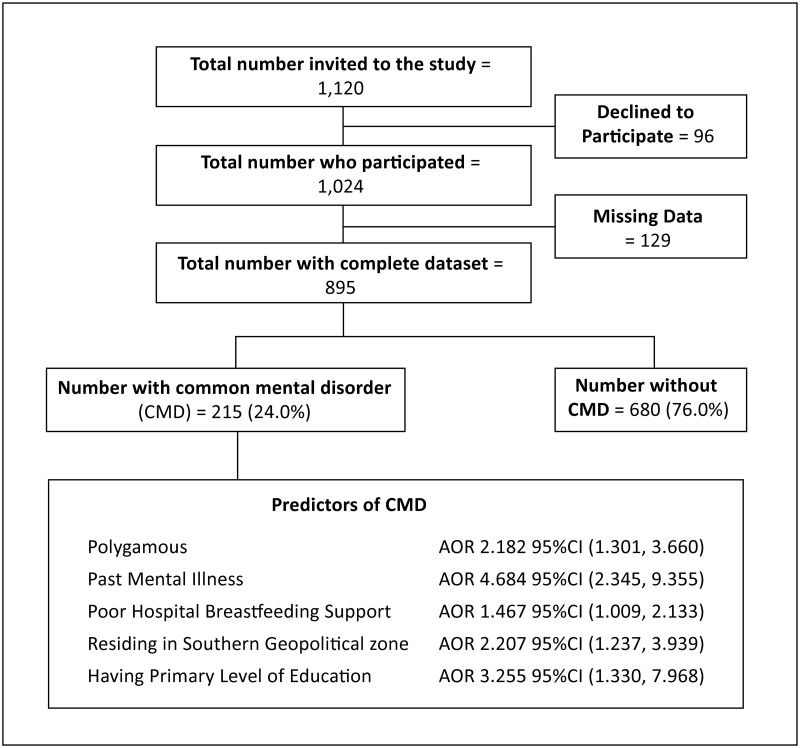
Participants flow chart study on common mental disorders among breastfeeding mothers in Nigerian tertiary hospital nurseries (The flow chart describes the participants’ enrollment and the predictors of common mental disorders among breastfeeding mothers whose babies were admitted to tertiary nurseries).

Eleven of 42 hospitals were selected as study sites utilising a multistage sample technique and GraphPad Prism 9 (GraphPad Software, 2365 Northside Dr Suite San Diego, CA 92108), (Fig 1.) Map of Nigeria showing selected Nigerian tertiary hospital nurseries in the six geopolitical zones; [UCH-University College Hospital, JUTH- Jos University Teaching Hospital, RSUTH-Rivers State University Teaching Hospital, FMC-Katsina: Federal Medical Centre, Kastina. ATBUTH-Abubakar Tafawa Balewa Teaching Hospital, Bauchi, UNTH-University of Nigeria Teaching Hospital, FMC-Lokoja; Federal Medical Centre, Lokoja, NUTH-Nile University Teaching Hospital Abuja; (formerly Asokoro District Hospital), LASUTH-, Lagos State University Teaching Hospital, NAUTH-Nnamdi Azikiwe University Teaching Hospital]. (Fig 2. Participants flow chart study on common mental disorders among breastfeeding mothers in Nigerian tertiary hospital nurseries).

Using stratified lists of hospitals per geopolitical zone, we selected one tertiary health centre per zone, for a total of six, using simple random sampling. In the second stage, we utilised a simple random procedure to select five additional tertiary health institutions from the pooled lists of tertiary health facilities across the country, bringing the total number of research sites to eleven. Using the approach of convenience sampling, study participants at each site were selected by picking all eligible consecutive admissions. At each study site, the co-investigator or a trained research assistant administered the Google Form to pairs of eligible mothers and infants in person. However, one of the centres in Northwestern Nigeria was inactive and was therefore excluded from the final data analysis. An inactive centre in Northwestern Nigeria was however, excluded from the data analysis.

### Study tools

The self-reporting questionnaire (SRQ)-20 of the World Health Organization (WHO) was used to evaluate maternal mental health. It is a 20-item questionnaire that inquires about depressive, anxious, panic, and somatic symptoms in the four weeks prior. Each of the twenty items received a score of 1 or 0 to indicate the presence or absence of symptoms. The measure ranged from 0 to 20, with higher scores indicating poor mental health and lower values indicating good mental health [20]. As suggested by many studies, a cut-off of 7 was used to classify women as having common mental disorders (CMD)’ or no CMD [3, 20–22]. The scale was administered in English. Collaborating investigators from the geopolitical zone translated the questionnaire into the major local dialects, face-validated it, and requested that it be reviewed by Psychometricians in regions with low levels of illiteracy. This instrument’s reliability and validity are well established, and it has been used in local studies in Nigeria [11].

### Other components of the questionnaire

The other components of the questionnaire included sociodemographic information, antenatal care, and questions about breastfeeding support. (hospital and family). The questions on breastfeeding support were derived following proper appraisal of relevant literature, WHO and UNICEF packages on successful breastfeeding [23]. Experts and members of the research team evaluated the derived breastfeeding questions and made additional modifications. Before the study commenced, we pilot-tested and validated the questionnaire with 90 mothers-dyads in one of the tertiary nurseries in southwestern Nigeria. The mother-baby pairs who took part in the pilot study were excluded from the final study. The pilot testing feedback was incorporated into the study’s final questionnaire. The details in the questionnaire can be accessed using the link: https://forms.gle/BJmWKdjkzDdt47oXA.

### Data management and analysis

The data were collected via Google form, extracted and exported into the Statistical Package for Social Sciences (S.P.S. S) version 23 for analysis. Sociodemographic factors, parity, and details of ANC, including gestational age at delivery, are summarised in the frequency table. Using a cut-off of 7, the SR1-20 was used to classify the mothers into CMDs and no CMDs groups. The mean score of the response was used to classify breastfeeding support as good (values corresponding to a mean score of 11.5 ± 5.2 and above) or poor (values below the mean score of 6.3). Pearson’s chi-squared test was used to examine associations between CMD and sociodemographics, gestational age at delivery, parity, baby’s duration on admission, and breastfeeding support while Fischer’s exact test was used to test for association between CMDs and antenatal factors (ANC attendance and place). For the Oyedeji social class, the education and occupation/business engagement of both parents were stratified into five categories. Parental occupations range from unemployed to senior public servants, professionals, managers, large-scale traders, businessmen, and contractors, while educational attainment ranges from no formal education to a graduate degree. The mean score of both parents at each stratum was estimated to the nearest integer, and a score was assigned. Furthermore, the five grades are reclassified into three social classes (upper: social classes I & II, middle: social classes III, and low: classes IV & V) [24]. We defined a monogamous family as a mother in an exclusive conjugal relationship with her partner, as do single mothers’ perceptions of being in an exclusive relationship with her partner before separation.

A mother is considered to have a polygamous marital status if she is in a relationship with a man who has or is having concurrent conjugal relationships with other women. Multivariable analysis was used to determine and report the adjusted odds ratio (AOR) with a 95% confidence interval for predictor variables that were statistically associated with CMDs on bivariable analysis. The predictor variables include geopolitical zones, educational level, location of ANC, marital status, family settings, and past mental illness. The significance level was set at *p* < 0.05.

### Ethical considerations

The National Health Research Ethics Committee of Nigeria granted ethical permission (NHREC/01/01/2007-31/03/2022) (NHREC) for the study. This study was conducted in accordance with the Helsinki Declaration. Before enrollment, voluntary participation was required of invited study participants. The details of the study were explained to the respondents, who were then asked to describe the study, and their comprehension was assessed. The respondent then voluntarily provided electronic consent by selecting yes to continue the questionnaire or no to decline participation. The data was redacted and password-protected to ensure privacy. Mothers found to have common mental disorders were referred to a psychiatrist and/or a psychologist for intervention.

## Results

Of the 1,120 mothers-dyads recruited for the study, at the tertiary healthcare nurseries in the six geopolitical zones in Nigeria, 895 (79.9%) had a complete dataset for analysis. Fig 2 shows the flow diagram of the study participant. The mean age of the study participants was 29.9 ± 6.2 years, and 76.2% of them were between the ages of 25 and 40. The majority of the mothers 835 (93.3%) received antenatal care, and approximately half were para 2 to 4 (48.5%). Nearly two-thirds (547, 61.1%) of the infants were born at term. Even though only 9.8% of participants lacked formal education, more than half, 490 (54.7%) belonged to a lower socioeconomic class. The socio-demographic data in Table 1 provide the details.

**Table 1:** General characteristics of the study population.

Variables	Subcategory	Frequency	Percent
		n = 895	
**Age group (years)**	15–20	69	7.7
	>21–<34	597	66.7
	≥35	229	25.6
**Parity**	1	339	37.9
	2–4	434	48.5
	≥5	122	13.6
**ANC**	No	60	6.7
	Yes	835	93.3
**Place of ANC**	Maternity home	14	1.7
	Private hospital	44	5.3
	Primary health facility	267	32.0
	Secondary health facility	221	26.5
	Tertiary health facility	289	34.5
**Gestational age at delivery**			
	Preterm	304	34.0
	Term	591	66.0
**Baby’s duration on admission (days)**	1–3	208	23.2
	4–7	386	43.1
	>7	301	33.6
**Educational level**	No formal education	88	9.8
	Primary	44	4.9
	Secondary	314	35.1
	Tertiary	449	50.2
**SEC**	Upper	127	14.2
	Middle	278	31.1
	Lower	490	54.7
**Geopolitical zones**	North-west	124	13.9
	North-east	75	8.4
	North-central	281	31.4
	South-east	93	10.4
	South-south	103	11.5
	South-west	219	24.5
**Marital status**	Married	854	95.4
	Single	41	4.6
**Family settings**	Monogamous	779	87.0
	Polygamous	116	13.0

ANC-antenatal clinic attendance, SEC-Socioeconomic class Mean age 29.9 ± 6.2

**Fig 3:**
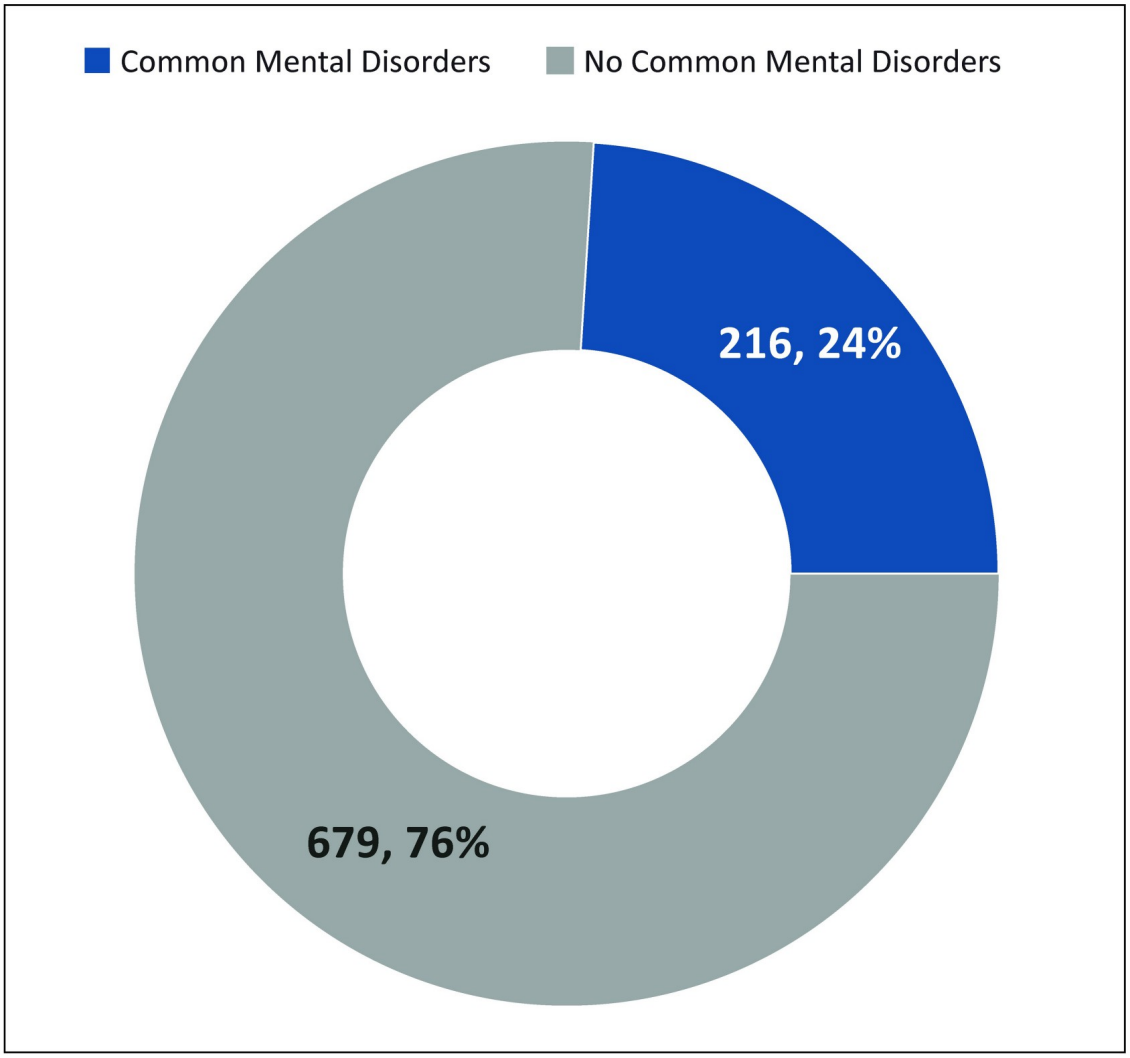
Prevalence of common mental disorders.

Common mental disorders were present in one in four; 24.0% (95% CI: 21.235, 26.937%) of breastfeeding mothers in tertiary hospital nurseries (Fig 3. Prevalence of Common Mental Disorders (CMDs)).

Overall, less than half (427; 47.7%) of the mother received optimal breastfeeding support in the hospital. Only 198 (22.1%) breastfeeding mothers received family support for breastfeeding while nursing babies in the hospital nursery. Of the 895 respondents, 41 (4.6%) disclosed having been diagnosed with mental health disorders.

The ages of mothers with CMDs and mothers without CMDs were comparable. Similarly, parity, gestational age at delivery, length of hospital stay, socioeconomic status, hospital breastfeeding support, and family support were comparable between the two groups (Table 2). In contrast, there were significant differences in the place of antenatal care attendance, educational levels, geopolitical zones within the country, marital status, family settings, and background history of mental disorders between mothers with CMD and those without CMD. (Table 2).

**Table 2:** Factors associated with common mental disorders (CMDs).

Variables	Subcategory	CMDs	No CMDs	χ2	P
	n = 895	n = 216	n = 679		
**Age group**	15–20 (69)	17(24.6)	52(75.4)	0.056	0.967
	>20–<35 (597)	145(24.3)	452(75.7)		
	≥35(229)	54(23.6)	175(76.4)		
**Parity**	1 (339)	77 (22.7)	262 (77.3)	0.615	0.735
	2–4 (434)	109 (25.1)	325 (74.9)		
	>4 (122)	30 (24.6)	92 (75.4)		
**ANC**	None (60)	20 (33.3)	40 (66.7)	22.225f	<0.001
	Maternity home (14)	5 (35.7)	9 (64.3)		
	Private hospital (44)	12 (27.3)	32 (72.7)		
	Primary (267)	79 (29.6)	188 (70.4)		
	Secondary (221)	57 (25.8)	164 (74.2)		
	Tertiary (289)	43 (14.9)	246 (85.1)		
**Gestational age**	Preterm (304)	226(74.3)	78(25.7)	0.586	0.459
	Term (591)	453(76.6)	138(23.4)		
**Baby’s admission duration**	< 3 (208)	61 (29.30	147 (70.7)	4.029	0.133
	3–7 (386)	86 (22.3)	300 (77.7)		
	>7 (301)	69 (22.9)	232 (77.1)		
**Educational level**	No formal education (88)	18 (20.5)	70 (79.5)	13.618	0.003
	Primary (44)	20 (45.50	24 (54.5)		
	Secondary (314)	81 (25.8)	233 (74.2)		
	Tertiary (449)	97 (21.6)	352 (78.4)		
**Socioeconomic class**	Upper (127)	37 (29.1)	90 (70.90	3.891	0.143
	Middle (278)	57 (20.5)	221 (79.5)		
	Lower (490)	122 (24.9(	368 (75.1)		
**Geopolitical zones**	Northwest (124)	18 (14.5)	106 (85.5)	17.840	0.003
	Northeast (75)	21 (28.0)	54 (72.0)		
	Northcentral (281)	62 (22.1)	219 (77.9)		
	Southeast (93)	30 (32.3)	63 (67.7)		
	Southsouth (103)	36 (35.0)	67 (65.0)		
	Southwest (219)	49 (22.4)	170 (77.6)		
**Marital status**	Married (854)	198 (23.2)	656 (76.8)	9.171	0.002
	Single parent (41)	18 (43.9)	23 (56.1)		
**Family settings**	Monogamous (779)	178 (22.8)	601 (77.2)	5.414	0.020
	Polygamous (116)	38 (32.8)	78 (67.2)		
**Hospital Breastfeeding support**	Poor (435)	112 (25.7)	323 (74.3)	1.203	0.273
	Good (460)	104 (22.6)	356 (77.4)		
**Family Breastfeeding support**	Yes (198)	55 (27.8)	143 (72.20	1.844	0.175
	No (697)	161 (23.1)	536 (76.9)		
**Past Mental illness**	Yes (41)	24 (58.5)	17 (41.5)	27.775	<0.0001
	No (854)	192 (22.5)	662 (77.5)		

ANC-antenatal care; f Fischer’s exact test

In the multivariable analysis, factors that were significantly associated with CMDs included the place of ANC attendance, with primary healthcare facilities having up to 13 times the odds for CMDS. The primary level of education (adjusted odds ratio [aOR] 3.255, 95% CI 1.330, 7.968), living in the south-southern region of the country [aOR 2.207, 95% CI: 1.237, 3.939], poor breastfeeding support [aOR 1.467, 95% CI 1.009, 2.133], polygamous family settings [aOR 2.182, 95% CI 1.301, 3.660], and a previous history of mental health disorders [aOR 4.684, 95% CI 1.301, 9.355]. In contrast, those from the middle and lower socioeconomic classes were less likely to develop CMDs, with [aOR 0.532] and [aOR 0.493] respectively (Table 3).

**Table 3:** Multivariable logistic regression of factors associated with CMDs.

Variables	CMDs	OR	95% CI	Adjusted OR	95% CI	P
	n = 216					
**Place of ANC**						
Tertiary	43 (19.9)	1				
Secondary	57 (26.4)	1.988	1.277, 3.095	4.664	2.200, 9.888	<0.001
Primary	79 (36.6)	2.404	1.584, 3.648	3.749	1.094, 12.848	0.035
Private hospital	12 (5.6)	2.145	1.025, 4.489	2.372	1.061, 5.306	0.035
Maternity home	5 (2.3)	3.178	1.016, 9.940	2.627	1.602, 4.306	0.001
None	20 (9.2)	2.860	1.528. 5.354	2.201	1.364, 3.533	0.001
**Baby’s duration on admission (days)**						
< 3	61 (28.2)	1				
3–7	86 (39.8)	0.691	0.471, 1.013	0.776	0.501, 1.202	0.256
>7	69 (32.0)	0.717	0.480, 1.071	0.754	0.479, 1.187	0.223
**Educational level**						
No formal edu.	18 (8.3)	1				
Primary	20 (9.3)	3.241	1.474, 7.124	3.255	1.330, 7.968	0.010
Secondary	81 (37.5)	1.352	0.760, 2.406	1.538	0.743, 3.183	0.246
Tertiary	97 (44.9)	1.072	0.609, 1.885	1.358	0.621, 2.970	0.443
**SEC**						
Upper	37 (17.1)	1				
Middle	57 (26.4)	0.627	0.388, 1.015	0.532	0.316, 0.896	0.018
Lower	122 (56.5)	0.806	0.522, 1.245	0.493	0.286, 0.849	0.011
**Geopolitical zones**						
Northcentral	62 (28.7)	1				
Northeast	21 (9.7)	1.374	0.771, 2.447	0.972	0.493, 1.915	0.934
Northwest	18 (8.3)	0.600	0.338, 1.065	0.497	0.257, 0.960	0.037
Southeast	30 (13.9)	1.682	1.002, 2.824	1.372	0.760, 2.475	0.494
Southsouth	36 (16.7)	1.898	1.159, 3.109	2.207	1.237, 3.939	0.007
Southwest	49 (22.7)	1.018	0.666, 1.557	0.777	0.483, 1.249	0.297
**Hosp BF sup**.						
Good	104 (48.1)	1				
Poor	112 (51.9)	1.187	0.874, 1.613	1.467	1.009, 2.133	0.045
**Marital status**						
Married	198 (91.7)	1				
Single	18 (8.3)	2.593	1.371, 4.902	1.979	0.986, 3.971	0.055
**Family settings**						
Monogamous	178 (82.4)	1				
Polygamous	38 (17.6)	1.645	1.078, 2.509	2.182	1.301, 3.660	0.003
**Family BF sup**						
No	161 (74.5)	1				
Yes	55 (25.5)	1.280	0.896, 1.831	1.189	0.787, 1.796	0.412
**Past Mental illness**						
No	192 (88.9)	1		1		
Yes	24 (11.1)	4.868	2.562, 9.248	4.684	2.345, 9.355	<0.001

CMDs-common mental disorders; OR-odds ratio; CI-Confidence intervals; ANC-Antenatal care; edu-education; SEC-socioeconomic class; BF sup-breastfeeding support

## Discussion

Common mental disorders refer to a range of nonpsychotic mental health conditions, such as anxiety, depression and somatoform disorders. In this study, the prevalence of CMDs among breastfeeding mothers in tertiary hospital nurseries in Nigeria was 24%. This demonstrates that one in four of the women in this study had CMDs. The additional strain of having a child in the nursery may be the cause of this. Furthermore, several studies have suggested that the challenges of nursing may increase the frequency and/or severity of maternal psychological disorders [25, 26].

The prevalence of CMDs in the mothers in this study is low when compared to the prevalence of CMDs among other categories of mothers in other studies. Among pregnant women, a higher prevalence range of 35.8% to 39.5% has been documented [27–31], while the documented range among mothers of children under the age of five years was 36.6% to 46.2% [32–34]. The low prevalence of CMDs among BF mothers may be due to the wide range of psychological benefits of breastfeeding to the mother. It has been documented that the act of breastfeeding promotes hormonal processes that induce the release of oxytocin, an important hormone related to maternal bonding, and attenuates the cortisol response to stress, which when consistently high, is one of the strongest risk factors for the development of psychiatric disorders [35]. Moreover, breastfeeding supports the regulation of sleep and wake patterns for both mother and infant and sustains and improves maternal self-efficacy. Consequently, breastfeeding mothers are more likely to report positive mood, less anxiety, and increased calm compared to formula-feeding mothers [36, 37]. Furthermore, Yeun *et al*., [38] in a systematic review of 36 studies, concluded that breastfeeding was associated with improved maternal mental health outcomes.

Previous studies on maternal mental health in Nigeria and other low-income countries found a much lower prevalence [39–41]. This disparity may be due to differences in the morbidity spectrum targeted, as well as differences in the sensitivity of measuring instruments and their cut-off points for mental health disorders [41]. This disparity may also be explained by differences in the timing of assessments during pregnancy and postpartum. It was also suggested that lower CMDs observed among childbearing women in high-income countries could be attributed to better maternal support during pregnancy and the immediate postnatal period than women in low-income countries [41].

Regarding the associated factors, place of ANC, level of education, place of abode, poor breastfeeding support, polygamous family setting and history of mental illness were significantly associated with CMDs. Mothers who attended ANC in PHCs were 13 times more likely to develop CMDs than those who received ANC in other facilities. This could be traceable to the quality of care received and the level of competence of the health workers in PHC. According to Ntoimo et al., [42]. pregnant women were hesitant to attend ANC in PHC due to the mothers’ palpable stress caused by a poor road network, difficulty with transportation, long distances, and random facility openings. The use of abusive care by healthcare professionals, the severe shortage of healthcare professionals in these facilities, the lengthy wait times, and the discouragement of partner support are other reasons to mention a few [42]. These possible stressors could predispose pregnant women attending ANC in this facility to CMD. Attending antenatal clinics has also been linked to an increased understanding of pregnancy complications, including mental diseases, as well as increased chances of learning about pregnancy readiness and reducing risk factors [43, 44]. This highlights the need for the government and private individuals to restructure PHC with qualified personnel and improved infrastructure, as well as a shift in the attitudes of health workers toward pregnant women. In contrast, pregnant women at high risk of CMDs should make a secondary health facility their first point of contact. Women with poor or no educational attainment are susceptible to CMDs [14, 34]. In the current study, mothers with primary education were three times more likely to have CMDs. Therefore, the educational level has a major impact on maternal mental health as higher education was found to be protective against maternal mental disorders [41, 45]. According to this study, mothers who dwell in the south of the country are twice more likely to exhibit CMD symptoms than mothers who live in the north. The reason for the regional variation in CMDs in the present study is unclear and may necessitate further research. However, published literature suggests that the variation may be due to differences in obstetric care [41]. Regions with a low tolerance for mother-baby separation and early, close relative access to the mother after birth will have better coping with perinatal stress and, as a result, a lower prevalence of CMDs [46]. In addition, the disparity may be attributed to variations in neonatal intensive care settings. In settings where family members have limited access to mother-baby dyads, the enormous benefits of family-centred care including mental health support from several reports are undermined [41, 47, 48]. Again, the importance of regional sociocultural differences in influencing mothers’ mental health cannot be overstated. In a special WHO report on maternal mental health in low- and middle-income countries, sociocultural factors such as breastfeeding not being fashionable and, for cosmetic preservation of breast shape, the class perception of the feeding bottle, all of which may deprive the mother of the mood-enhancing effect of breastfeeding, are cited as risk factors for CMDs [41]. In addition, the cultural perception of a mother’s breastfeeding difficulties as a sign of weakness by family members increases the mother’s stress level and predisposes her to mental disorder.

Polygamous household settings were two times more likely to have CMDs in the current study similar to finding in northern Nigeria by Imam *et al*., [49] and A WHO report on CMDs in LMIC [41]. This might be because polygamy comes with additional pressures. In contrast, Abdullahi *et al*., [11] found no evidence of polygamy as a risk factor for CMDs because the practice was prevalent in the study settings and was regarded as the norm [11].

Notably, this study also found a correlation between a prior history of mental illness and CMDs among nursing mothers, with an odds ratio as high as 4.684. This finding supported the literature’s contention that past mental health issues increase the chance of developing postpartum mental illnesses in the future to as high as 22 folds [7, 45, 50, 51]. This highlights the importance of upscaling mental health services as a core component of antenatal and postnatal care, particularly in low-income countries where such services are limited. Our observation of no apparent relationship between maternal age and CMDs is in contrast to other research findings, which showed a substantial relationship between CMDs and higher maternal age [13, 14]. Perhaps the predominance of younger mothers in our study population helps to explain the lack of an association between age and maternal CMDs in the current study. In addition, the SRQ-20 was not designed to detect psychotic disorders which are linked to older age groups.

### Study limitations

Although this study had a good sample size involving all of the major regions of Nigeria, the number of voluntary participants differed across geopolitical zones, with the southeastern region of Nigeria having the lowest number of individuals. This survey was also cross-sectional in design; a prospective study with adequate follow-up would have revealed more about the maternal level of CMD. However, we hope that this will serve as a basis for a more robust longitudinal study design utilising validated instruments to help define the specific mental disorders among breastfeeding mothers with infants admitted to tertiary facilities for targeted intervention. We hope that the second phase of this study will take a longitudinal design approach, assuming that funding will be available.

## Conclusion

In Nigeria, the prevalence of common mental disorders is relatively high among breastfeeding mothers with infants admitted to a tertiary care facility. Populations with a history of mental illness, polygamous households, mothers living in the southern region of the country, and low or no educational attainment have a greater risk of developing common mental disorders. This study provides evidence for assessing and tailoring interventions to this specific population.

## Supporting information

S1 Appendix PlosOne dataset factors associated with CMD 20082022.sav 2 (6) (1). (SAV)
